# Integrated Bulk and Single-Cell Transcriptomics Reveals the C3–C3AR1 Axis as a Candidate Mediator of Coagulome-Immune Crosstalk in Osteosarcoma

**DOI:** 10.3390/biomedicines14081686

**Published:** 2026-07-27

**Authors:** Jianhua Mu, Yitian Wang, Han Liu, Xuanhong He, Zhuangzhuang Li, Minxun Lu, Fan Tang, Yi Luo, Yong Zhou, Li Min, Chongqi Tu

**Affiliations:** 1 Department of Orthopedics, Orthopedic Research Institute, West China Hospital, West China Medical School, Sichuan University, Chengdu 610207, China; 2 Model Worker and Craftsman Talent Innovation Workshop of Sichuan Province, West China Hospital, Sichuan University, Chengdu 610207, China

**Keywords:** single cell, osteosarcoma, macrophage, coagulome, immune crosstalk

## Abstract

**Background:** Although the tumor coagulome interacts with the tumor immune microenvironment (TME) in solid tumors, its role in osteosarcoma (OS) remains uncharacterized. This study aimed to delineate this transcriptomic interplay and identify potential prognostic targets. **Methods:** This study was performed with bulk RNA sequencing (RNA-seq), single-cell RNA sequencing (scRNA-seq), and clinical phenotype data. Bioinformatic approaches were employed at the transcriptomic level to investigate the impact of the tumor coagulome on the TME and prognosis in OS. We validated the above findings using immunohistochemistry and immunofluorescence. **Results:** The activity of a coagulation-related transcriptional signature was found to correlate with the degree of malignancy in OS. Its activity score demonstrated predictive value for OS prognosis, with a maximum area under the curve (AUC) of 0.802. scRNA-seq analysis indicated that inflammatory cancer-associated fibroblasts (iCAFs) and APOE^+^ macrophages were predominantly enriched in the high coagulation score subgroup. Our data further suggest that iCAFs may facilitate the M2 polarization of APOE^+^ macrophages via the C3–C3AR1 axis, potentially contributing to poorer clinical outcomes in patients with OS. **Conclusions:** These findings imply that within a high coagulation-related transcriptional score group, the interaction between iCAFs and APOE^+^ macrophages, likely mediated by the C3–C3AR1 axis, could facilitate OS progression. Consequently, the C3–C3AR1 signaling pathway might represent a promising target for future therapeutic strategies and coagulome monitoring in OS.

## 1. Introduction

Osteosarcoma (OS) is a highly prevalent malignant tumor in adolescents, characterized by its aggressive nature and poor prognosis [1]. The prognosis for OS patients has markedly improved in recent years through combined neoadjuvant chemotherapy, targeted therapy, and immunotherapy, with 5-year survival rates rising from historically 20% to the current 75% [2,3]. This breakthrough is closely related to the deepened understanding of OS pathogenesis and the tumor microenvironment (TME).

The tumor coagulome is a molecular effector network driven by cancer, participating in tumor progression by regulating thrombotic or hemorrhagic events [4]. Evolutionary biology studies suggest that hemocytes in early organisms may represent a common origin for the development of the immune and coagulation systems [5]. This association is manifested in the TME as a complex interplay between the tumor coagulome and the TME. For instance, tumor-associated macrophages (TAMs), as key components of the TME, can promote coagulation by secreting coagulation factor X (FX) and activating the FXa-PAR2-signaling pathway [6,7]. Concurrently, coagulation-related molecules, such as plasminogen activator inhibitor-1 (PAI-1) and thrombin, can regulate the M2 polarization of TAMs in lung and ovarian cancer, respectively [8,9], which reveals a bidirectional regulatory mechanism between the coagulation system and TME. Furthermore, increasing attention is being paid to the impact of the tumor coagulome on treatment response. Preclinical studies have confirmed that the direct oral anticoagulant (DOAC) rivaroxaban can enhance the efficacy of immune checkpoint blockade (ICB) in malignant melanoma [10]. Coagulation-related biomarkers have also been proven to have prognostic predictive value in various solid tumors, including liver cancer [11], breast cancer [12], and glioma [13], melanoma [14], gastric cancer [15], and ovarian cancer [16]. Although coagulation-related biomarkers have been found to predict patient prognosis in OS [17], research remains scarce, which elucidates the mechanism of the tumor coagulome in OS at the transcriptomic level.

The breakthrough of single-cell RNA sequencing (scRNA-seq) technology has provided a novel perspective for in-depth analysis of the TME. Zhang’s team, by integrating pan-cancer single-cell atlases, has systematically depicted the cross-cancer heterogeneity of T cells [18], B cells [19], myeloid cells [20], and NK cells [21], which provides a theoretical framework for exploring the functional landscape of the TME. Furthermore, based on these research findings, there is immense potential for analyzing the mechanisms of the tumor coagulome and its interaction with the TME in OS at the single-cell dimension.

Here, we leveraged publicly available bulk and single-cell transcriptomic datasets to perform multi-layered bioinformatic analyses, which were subsequently validated against institutional clinical profiles and preliminary histological matrices. Our results suggest that, in a high coagulation-related transcriptional score group, inflammatory cancer-associated fibroblasts (iCAFs) may promote the M2 polarization of APOE^+^ macrophages via the C3–C3AR1 axis, potentially contributing to OS progression. Furthermore, C3AR1 emerges as a candidate pathogenic gene in OS with possible roles in other malignancies, highlighting a prospective target for TME-directed combination therapies.

## 2. Materials and Methods

### 2.1. Public Data Acquisition

Transcriptome data involved in this study are collected from public data sets. The microarray data of OS samples include GSE21257 from the Gene Expression Omnibus (GEO) database (Gene Expression Omnibus. https://www.ncbi.nlm.nih.gov/geo/. Accessed on 20 May 2025.) [22] and TARGET-OS from the bulk RNA-seq TARGET database (https://xenabrowser.net/datapages/. Accessed on 20 May 2025.), while the bulk RNA-seq data of normal tissues are from the Genotype-Tissue Expression (https://www.genome.gov/Funded-Programs-Projects/Genotype-Tissue-Expression-Project. Accessed on 25 May 2025) [23]. scRNA-seq datasets of OS numbered GSE152048 [24] and GSE162454 [25] were obtained from GEO database. Firstly, the R package Seurat was used to filter cells using the following criteria: the number of expressed genes was less than 200 or more than 7500, and cells with more than 20% UMI mapped to mitochondrial genes were excluded. We retained only genes expressed in ≥5 cells. After that, the data were normalized, and the top 2000 highly variable genes were detected by “FindVariableFeatures” function [26]. Secondly, based on 2000 genes, the dimension of scRNA-seq data was reduced by principal component analysis (PCA), and 40 principal components were selected using the uniform manifold approximation and projection (UMAP) algorithm for subsequent analysis. Finally, cell clustering uses the function of “FindClusters”. In order to determine the cell type, we annotated the cells according to the CellMarker database [27] and the corresponding literature. Spatial transcriptomics analysis was conducted using the public 10x Visium dataset GSE299025 (GEO; accessed on 20 June 2026) [28]. Data preprocessing and quality control followed the same criteria as for scRNA-seq. PCA and UMAP were applied for dimensionality reduction and clustering (resolution = 0.5), and spatial spots were mapped onto H&E-aligned tissue sections. In addition, bulk RNA-seq data of OS were obtained from UCSC Xena (https://xena.ucsc.edu. Accessed on 30 May 2025.) database, numbered GDC TARGET-OS. Gene sets with ID of KEGG_COMPLEMENT_AND_COAGULATION_CASCADES (KEGG), MODULE_131 (Broad Institute), WP_COMPLEMENT_AND_COAGULATION_CASCADES (WikiPathways) were obtained from Molecular Signatures Database (MSigDB) database, and a complete set of coagulation gene data was obtained by intersection of the three gene sets [29]. Spatial transcriptomic analyses were performed to evaluate the spatial co-localization of *C3AR1* with macrophage-associated markers in osteosarcoma tissue sections using Seurat-based processing. Normalized spot-level expression matrices were extracted for *C3AR1*, *CD68*, and *APOE*, and binary positivity was defined using a threshold of expression > 0. CD68^+^APOE^+^ spots were designated as macrophage-enriched regions. Conditional probability analysis was then applied to quantify the spatial dependency of *C3AR1* signals within macrophage zones, defined as P(Macrophage|C3AR1^+^), calculated as the proportion of C3AR1-positive spots overlapping with CD68^+^APOE^+^ regions relative to all C3AR1-positive spots. Gene sets related to lipid metabolism, phagocytosis, tissue repair, immunoregulation, and immunosuppression were retrieved from the GeneCards database (https://www.genecards.org/; accessed on 22 June 2026), selecting genes with a relevance score greater than 10 for subsequent functional enrichment and pathway analysis [30].

### 2.2. Clinical Data Acquisition

The data used in this study came from the eligible OS patients in West China Hospital. Retrospective clinical data were derived from patients treated between 4 November 2019 and 22 September 2022. Patients were enrolled based on the following strict inclusion criteria: (1) a definitive histopathological diagnosis of high-grade primary conventional intramedullary osteosarcoma, established via pre-treatment core needle or open surgical biopsy, and independently verified by two senior musculoskeletal pathologists strictly adhering to the current World Health Organization (WHO) Classification of Tumors of Soft Tissue and Bone guidelines; (2) no history of other synchronous or metachronous malignancies; (3) complete clinical, laboratory, and follow-up records; and (4) formal consent for the use of personal medical information for scientific research.

The exclusion criteria were defined as follows: (1) secondary osteosarcomas, low-grade intramedullary or surface variants, or craniofacial and jaw lesions; (2) presence of severe systemic comorbidities or active systemic infections; or (3) incomplete baseline clinical data that failed to meet the quality requirements of this study. A total of 59 patients were included in this retrospective study. Clinical data were extracted from the electronic medical records system. Given the retrospective nature of the study and the use of anonymized data, the requirement for written informed consent was waived by the Institutional Review Board (IRB) of West China Hospital. Pre-operative hypercoagulability in our institutional cohort was defined as concurrent elevations in plasma D-dimer (>0.55 mg/L FEU) and Fibrinogen (>4.0 g/L) based on the running baseline laboratory reference ranges. All baseline hematological screenings were performed within 3 days prior to the initial biopsy or neoadjuvant chemotherapy. Patients with concurrent systemic infections, active thromboembolic events (DVT/PE), or those receiving prophylactic anticoagulation therapy were excluded to minimize confounding inflation. Crucially, tissues used for downstream IHC/IF validation were collected sequentially from primary surgical resections post-chemotherapy, establishing a clear temporal order relative to baseline hematological testing. The baseline table of patients included in the study is shown in Table 1.

### 2.3. Enrichment Analysis

Enrichment analysis was carried out to determine whether a series of biological processes defined a priori are enriched. Gene Ontology (GO) analysis was carried out using R-package clusterProfiler (version 4.2.2), which is a general enrichment tool for interpreting omics data. In order to ensure that there are enough genes for GO analysis [31], compared with differential expression analysis, we relaxed the standard of expression score. Genes expressed in at least 25% of the cells in the cluster were used for GO analysis. Only the first five enrichment biological process items of each cell type are shown in the analysis. Adjusted *p*-values < 0.05 were deemed significant.

### 2.4. Score Using the Specified Gene Set

The target gene set was obtained from published articles or public databases. For bulk RNA-seq data, the GeneSetCollection function in Gene set variance analysis (GSVA, version 1.50.0) package was used to preprocess the specified gene set. Since all the bulk RNA-seq data we used were in fragments per kilobase of exon model per million mapped fragments (FPKM) format, the target gene set score of each sample of bulk RNA-seq data was further calculated by using GSVA function and setting the parameter kcdf = “Gaussian”. For scRNA-seq data, using the AddModuleScore function of Seurat R software package to score each cell.

### 2.5. Abundance Evaluation of Immune Cells with Bulk RNA-Seq Data

Tumor immune microenvironment has an important influence on various biological behaviors of tumors and the curative effect of immunotherapy. It is essential to understand the crosstalk of immune cells in tumor immune microenvironment. Immuno-oncology biological research (IOBR) makes a comprehensive analysis of the estimation; TME deconvolution and feature construction of reported or user-built features are based on multi-omics data [32]. Notably, IOBR also provides batch analysis of these features and their correlation with clinical phenotype, long non-coding RNA (lncRNA) analysis, genomic characteristics, and characteristics of scRNA-seq data generation in different cancer environments. Here, we mainly use xCELL and estimating the proportion of immune and cancer cells (EPIC) of IOBR package to calculate immune infiltration.

### 2.6. Cell-Cell Communication Analysis

In order to visualize and analyze the cell–cell communication of our data, we used the “CellChat” package [33] for CellChat analysis. The feature of this package is that it can combine the intercellular ligand-receptor communication with the intracellular transcription factor expression to form the ligand-receptor-transcription factor axis (L-R-TF axis). At the same time, it also includes pathway activity analysis, which allows the analysis of receptor-cell pathway changes caused by communication between two specific cell types. We create a new CellChat object from the Seurat object. The cell type has been added to the CellChat object as cell metadata. CellChat identifies the differentially overexpressed ligands and receptors in each cell group, and associates each interaction with a probability value to quantify the communication between the two cell groups mediated by these signal genes. Significant interactions are identified on the basis of statistical tests, which randomly arrange the group labels of cells and then recalculate the interaction probability. The results were visualized through the netVisual_bubble function. In addition, the results of cell communication with different prognosis scores were compared and analyzed.

### 2.7. Prediction of Possible Transcriptional Regulatory Networks Using pySCENIC

Understanding the transcriptional regulatory network of each pathway will help us to intervene in the designated pathway more accurately and effectively. Reference documents were based on hg38__refseq-r80__10kb_up_and_down_tss.mc9nr.genes_vs_motifs.rankings.feather and motifs-v9-nr.hgnc-m0.001-o0.0.tbl (https://github.com/aertslab/pySCENIC. Accessed on 30 May 2025.) Reference [34] was used to conduct single-cell regulatory network reasoning and cluster analysis (SCENIC). The default parameters were used in the SCENIC workflow, with the original count matrix of all as input. We calculated the coexpression module and used GRNBoost to evaluate the weight between transcription factor (TFs) and its target gene and then used RcisTarget to identify TFs with direct target (regulator). Finally, AUCell was used to evaluate the activity of each regulator in each cell. The above three steps were mainly completed by pyscenic grn, pyscenic ctx, and pyscenic aucell functions.

### 2.8. Prediction of Prognosis by Machine Learning Method

To explore the prognostic predictive role of specific genes for patient outcomes, we built a prognostic model using the expression matrix of the specified genes. Thus, we used the recently published Mime package [35]. The Mime package enables comprehensive and convenient construction of machine learning models to predict the prognosis. Mime also has the potential to guide biomarker development and contributes to personalized medicine by bridging the gap between computational biology and cancer research. Although the author demonstrates the application of Mime using transcriptomic data, it can support other input data such as proteomics, radiomics, clinical biomarker data, and other numerical matrices containing patient clinical information, supporting multi-omics research. It integrates various classical algorithms, including random survival forests (RSF), least absolute shrinkage and selection operator (LASSO), gradient boosting machines (GBM), survival support vector machines (survival-SVM), supervised principal components (SuperPC), ridge regression, Cox proportional hazards partial least squares (plsRcox), CoxBoost, stepwise Cox, and Elastic Net (Enet). Among these, RSF, LASSO, CoxBoost, and stepwise Cox have dimension reduction and variable selection functions. This enables us to calculate the C-index for each cohort’s features obtained in the training queue, thereby evaluating the predictive performance of the specified variables.

To construct a robust coagulation-complement-related prognostic signature, we performed a least absolute shrinkage and selection operator (LASSO)-penalized Cox regression analysis within the GSE21257 training cohort (N = 53) utilizing the glmnet package. To avoid overfitting, 10-fold cross-validation was strictly implemented to determine the optimal tuning parameter (λ). The mathematical risk score formula was established via the linear combination of the gene expression levels weighted by their respective regression coefficients derived from the minimum λ (λ min):

$$\mathrm{Risk}\;\mathrm{Score}=\sum_{i=1}^{n}\beta_{i}\cdot \mathrm{Expression}(Gene_{i})$$

where *n* denotes the total number of selected prognostic genes, $\beta_{i}$ represents the regression coefficient of the i-th gene derived from the LASSO-Cox model, and $\mathrm{Expression}({Gene}_{i})$ signifies the normalized transcriptomic expression level of the corresponding gene in a given sample. Patients within both the training and the independent TARGET-OS validation cohorts (N = 84) were stratified into high-risk and low-risk groups based entirely on the median risk score threshold calculated exclusively from the training dataset, thereby preventing any potential informational leakage or validation bias.

### 2.9. Single Gene Pan-Cancer Analysis

The analysis of single gene pan-cancer is helpful to understand the pathogenic mechanism and specific role of a single gene in multiple tumors, thus guiding early clinical screening, diagnosis and treatment. TCGAplot provides a user-friendly interface for analyzing the multi-disciplinary data of TCGA pan-cancer types and uses visualization technology to enable users to explore the commonness and heterogeneity of various types of tumors. Specifically, several functions have been developed to perform pairwise/unpaired expression analysis, correlation analysis, survival analysis and user-defined function analysis [36].

### 2.10. Immunohistochemistry and Immunofluorescence

Formalin-fixed, paraffin-embedded human osteosarcoma tissue sections were cut at 5 µm thickness (Leica RM2235, Leica Biosystems, Wetzlar, Germany), baked at 65 °C for 2 h, deparaffinized in xylene, and rehydrated through graded ethanol (100%, 95%, 85%, 75%) to distilled water, then rinsed in PBS (pH 7.4) and PBST. Antigen retrieval was performed in an EDTA buffer (pH 9.0) in a microwave at medium-low power for 10 min followed by an additional 25 min, cooled to room temperature, and washed three times in PBS (5 min each). Endogenous peroxidase was blocked with 3% H_2_O_2_ for 10 min, and non-specific binding was blocked with normal goat serum (100 µL/section, 20 min, RT). For chromogenic IHC, sections were incubated overnight at 4 °C with anti-C3aR1 (GeneTex GTX114293, 1:200), washed, incubated with secondary antibody (1 h, RT), developed with DAB, counter-stained with haematoxylin (~3 min), dehydrated, cleared in xylene and mounted. For immunofluorescence, following the same deparaffinization and antigen-retrieval steps, slides were incubated overnight at 4 °C with anti-C3aR1 (1:200), washed, incubated with HRP-conjugated secondary antibody (30 min, RT), treated with fluorescent tyramide reagent (3–10 min) for covalent fluorophore deposition, followed by heat-mediated stripping, nuclei counterstained with DAPI (10 min, RT, protected from light), washed in PBS, mounted with antifade medium. The definition of high coagulation-related transcriptional score group in osteosarcoma is a group of patients whose average preoperative blood test results show elevated levels of D-dimer and fibrinogen.

### 2.11. Statistical Analysis

In our research, bioinformatics analysis and visualization were both carried out by R software (version 4.1.2). Pearson correlation and Spearman correlation were used for correlation analysis. The specific method is described in the results section and graphic legend. Statistical significance is defined as * *p* < 0.05, ** *p* < 0.01, *** *p* < 0.001, and *p* < 0.05 is considered to be statistically significant. For multiple hypothesis testing, the Benjamini–Hochberg method was used to adjust the *p* value.

## 3. Results

### 3.1. Coagulation-Related Transcriptional Characteristics Are Involved in Tumor-Promoting Pathways in OS and Can Lead to Poor Prognosis

**Figure 1:**
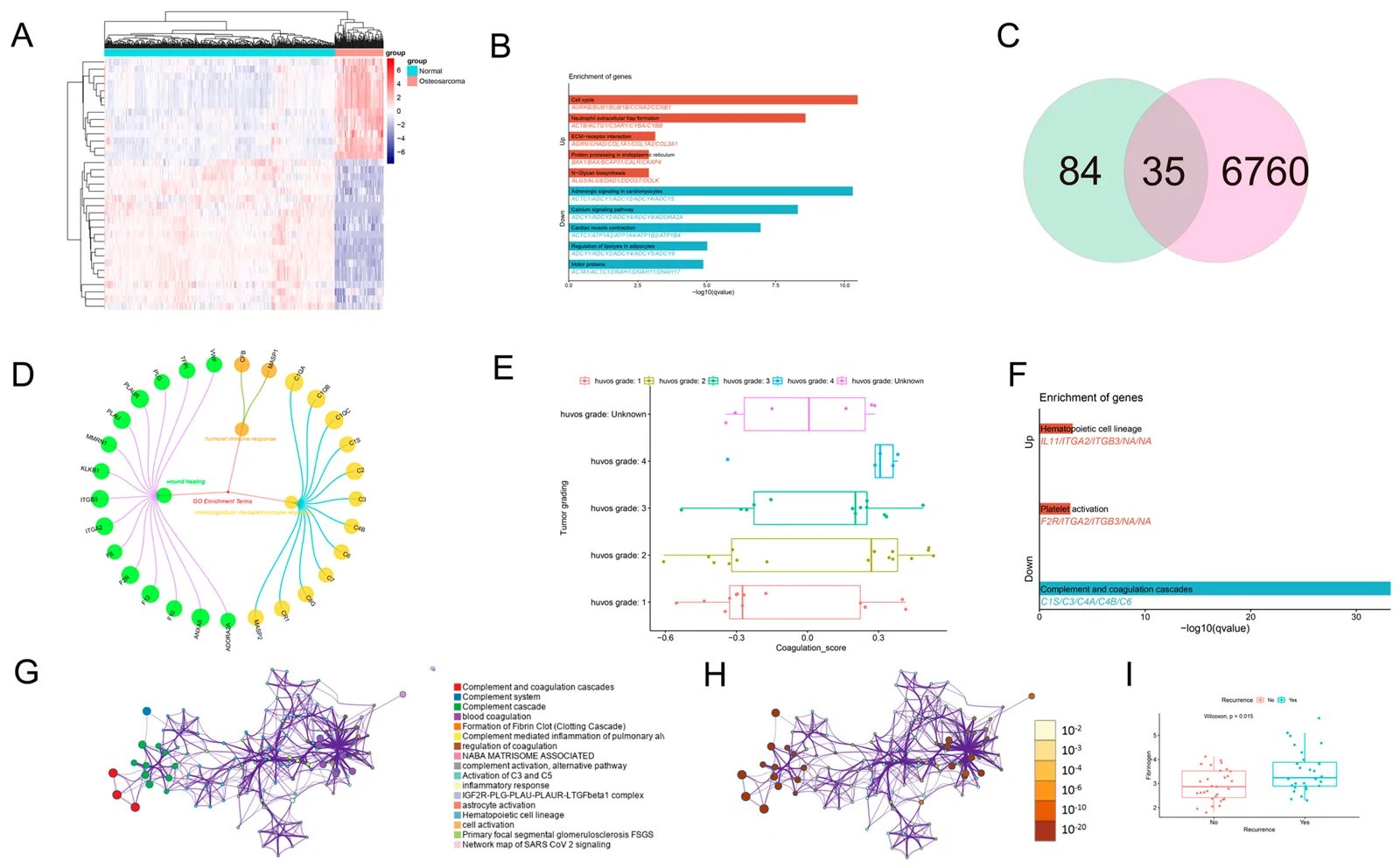
Differential gene expression and coagulation-related pathway enrichment in OS versus normal tissues. (**A**) Heatmap of differentially expressed genes between OS tissue and normal tissue. (**B**) Enrichment analysis results of differentially expressed genes in OS. (**C**) Intersection of differentially expressed genes in OS with coagulation-related genes. (**D**) Enrichment analysis of the 35 genes obtained from the intersection. (**E**) Scores of the 35 genes and their comparison among patients with different grades of OS. (**F**) Enrichment analysis of the 35 upregulated and downregulated genes. genes. (**G**,**H**) Network map of pathways involving the 35 genes, showing the weight of each pathway on the Metascape website. (**I**) Comparison of fibrinogen in patients with recurrent OS and primary OS.

Log(n + 1) normalization was applied to the bulk RNA-seq expression data from GEO, TARGET, and GTEx. In order to obtain the significantly different expression genes between OS samples and normal tissue samples, we used the “sva” package to remove the batch effect and obtained the expression matrices of 384 normal tissues and 84 OS samples. The ‘limma’ package was used for differential analysis with |logFC| ≥ 2 and *p* < 0.05 as significance thresholds. Finally, 6760 significantly differentially expressed genes were obtained, including 3347 up-regulated genes and 3413 down-regulated genes (Figure 1A). It can be found that the significantly different up-regulated genes in OS are mainly concentrated in the cell cycle and other pathways (Figure 1B). As is commonly known, abnormal cell cycle is an important cause of tumor occurrence, and it also implies that the occurrence of OS is related to abnormal activation of cell cycle-related pathways. In order to further understand the expression difference of coagulation-related transcriptome between OS and normal tissues, we collected 84 coagulation-related genes, obtained from the MsigDB database, and then intersected them with the significantly differentially expressed genes in OS and finally obtained 35 coagulation-related genes with significantly different expressions, of which 14 genes were differentially up-regulated in OS, and 21 genes were differentially down-regulated in OS (Figure 1C). Interestingly, these 35 genes are mainly enriched in immunoglobulin-mediated immune response, indicating that these genes may participate in the reprogramming of OS immune microenvironment (Figure 1D). GSVA was used to score the expression of coagulation-related genes in each OS sample according to the above differences. The results showed that there was a certain correlation between the grade of OS and the coagulation score; that is, the higher the score, the higher the grade of OS and the greater the relative malignancy (Figure 1E). The results of differential analysis show that platelet activation is the main enrichment pathway of 14 differentially up-regulated coagulation-related genes (Figure 1F). In recent years, platelets have been proved to dynamically interact with tumors and promote the survival and proliferation of tumor cells, which may also be the main pathway for the occurrence and progress of OS. The protein–protein interaction and action pathways of the above 35 genes were explored on the Metascape website. The results showed that multiple pathways of the complement and coagulation cascade, complement system, supplementary cascade, and blood coagulation played an increasing role in the protein–protein interaction (PPI) network (Figure 1G,H). Furthermore, analysis of 59 OS cases at the West China Hospital demonstrated that relapsed patients had markedly elevated coagulation profiles relative to non-relapsed patients (Figure 1I).

**Figure 2:**
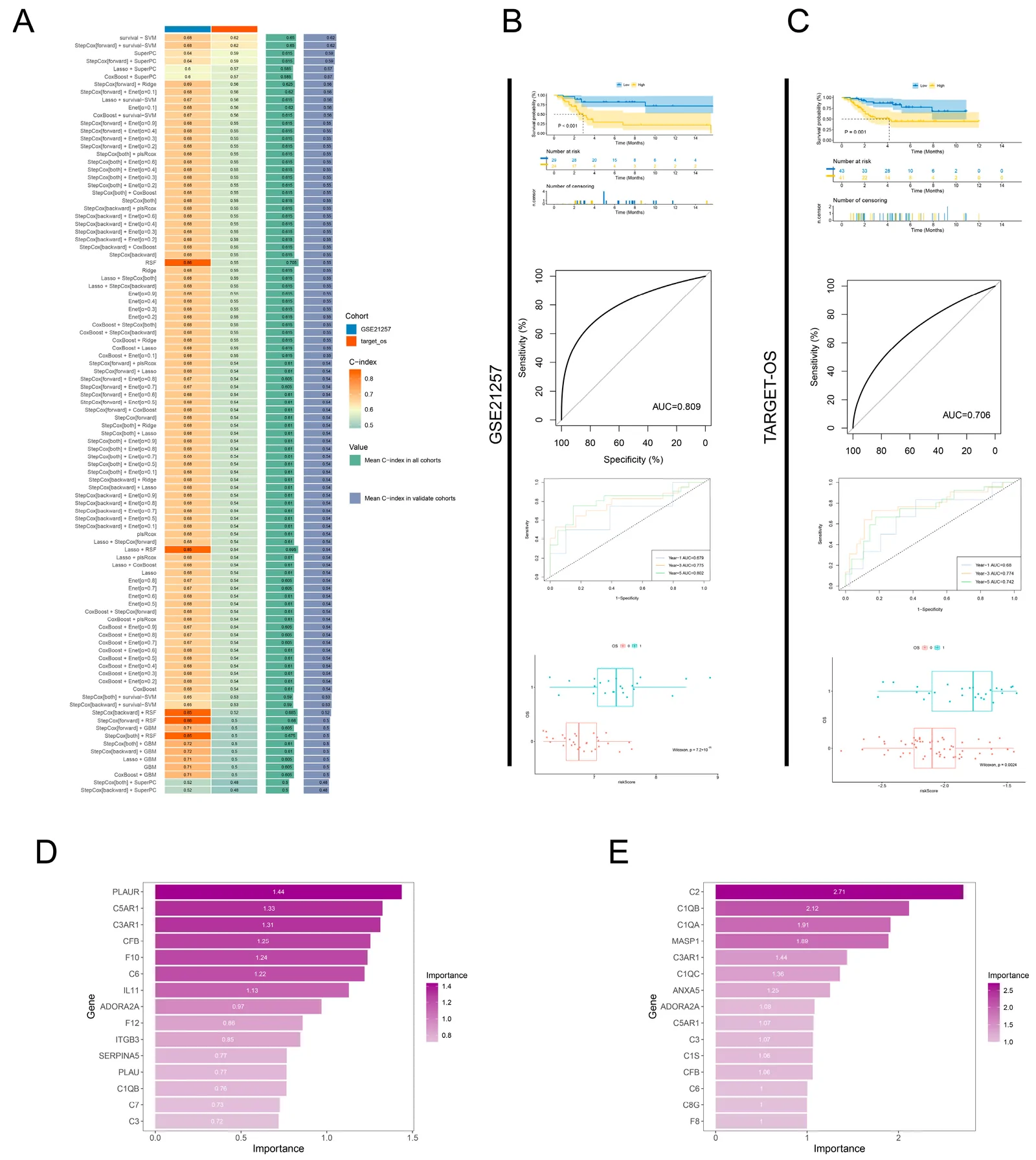
Prognostic validation of 35 coagulation-related genes in OS via LASSO and random forest models. (**A**) Exploring the prognostic role of 35 genes in OS patients using the Mime package. (**B**) Predicting the prognosis of 35 genes in the GSE21257 dataset using lasso regression. From top to bottom, K-M survival curves for patients in different coagulation risk groups; ROC curves for predicting prognosis based on coagulation scores; ROC curves for 1-year, 3-year, and 5-year predictions; comparison of coagulation scores across different outcomes. (**C**) Predicting the prognosis of 35 genes in the target OS dataset using lasso regression. (**D**) Quantifying the prognostic predictive power of 35 genes in the GSE21257 dataset using random forest methods, showing the top 15 most important genes. (**E**) Quantifying the prognostic predictive power of 35 genes in the target OS dataset using random forest methods, showing the top 15 most important genes.

Next, we explore the prognostic role of differentially expressed coagulation-related genes in patients with OS in transcriptomics. Firstly, the “Mime” package was used to explore the effect of different combinations of machine learning methods on the prognosis prediction of OS. The results showed that the survival-SVM and StepCox[forward]+ survival-SVM methods had the highest accuracy, reaching 0.68 and 0.62 in GSE21257 and TARGET-OS databases, respectively (Figure 2A). In order to further quantify the prognostic role of the above 35 genes, LASSO regression, which is commonly used in prognosis research at present, is used for analysis. The results show that the higher the prognosis score calculated by using 35 prognostic genes, the worse the prognosis of patients (Figure 2B,C). In addition, the gene score can maintain a certain accuracy over a long time duration. In both data sets, the 5-year survival prediction area under curve (AUC) exceeded 0.7, with increasing AUC values over time in the GSE21257 dataset. The gene score of dead patients was also significantly higher than that of non-dead patients. RSF analysis shows the detailed effect of each gene on prognosis, and it can be seen that *C3AR1* is the most significant gene in GSE21257 and TARGET-OS (Figure 2D,E, Supplementary Figure S1A,B).

### 3.2. Macrophages and Coagulation-Related Genes Play a Synergistic Role in Promoting Tumor

In order to further explore the tumor-related pathways involved and influenced by the coagulation-related transcriptome of OS, we used 35 coagulation-related genes and HALLMARKER50 data set obtained from MsigDB database, which is a gene set of 50 pathways most related to tumor progress integrated with a large number of basic experiments and related clinical studies. It can be found that the scores of GSE21257 and TARGET-OS are significantly consistent in most pathways, which shows that the heterogeneity of the two datasets is small and the abnormal pathways of OS are homogeneous (Supplementary Figure S2A). The score of IL-6-JAK-STAT3 pathway is high in the two data sets. Studies have shown that IL-6-JAK-STAT3 pathway is involved in some important biological processes, including cell proliferation, differentiation, apoptosis, immune regulation, and hematopoiesis [37]. In addition, patients with OS were scored by using the data set of tumor-related characteristics obtained by searching the literature and related databases, and the results showed that the inflammatory activity scored higher in the two data sets (Supplementary Figure S2B). In 1863, Virchow assumed that cancer originated in chronic inflammatory sites, which was partly based on his hypothesis that certain kinds of irritants, together with the tissue damage that caused the inflammation that followed, would enhance cell proliferation [38]. Therefore, in order to understand the characteristics of inflammatory cells and tumor immune microenvironment in OS, we used IOBR package to perform xCELL and EPIC methods on two OS data sets to get deconvolution immune cell scores (Supplementary Figure S2C,D). Mantel was used to evaluate the correlation between the ratio and abundance of immune cells and coagulation score in the immune microenvironment of OS, and it was found that there was a complex correlation between immune cells (Supplementary Figure S2E,F). Interestingly, there is a significant positive correlation between the cell abundance of macrophages and macrophage subtypes and the coagulation gene score, which is consistent in the two OS data sets (Supplementary Figure S2G,H). These changes in the phenotype and function of macrophages can lead to maladaptive repair, and lead to the development of chronic inflammation and pathological fibrosis [39]. These results indicate that macrophages may promote tumor progression by mediating chronic inflammation. Crucially, multivariable Cox analysis demonstrated that both coagulation score and macrophage abundance serve as significant, independent predictors of poor overall survival, with the coagulation score remaining highly robust across the GSE21257 (HR = 1.12, 95% CI: 1.01–1.24, *p* = 0.031) and TARGET-OS cohorts (HR = 1.15, 95% CI: 1.04–1.27, *p* = 0.009; macrophage: *p* = 0.041) (Supplementary Tables).

**Figure 3:**
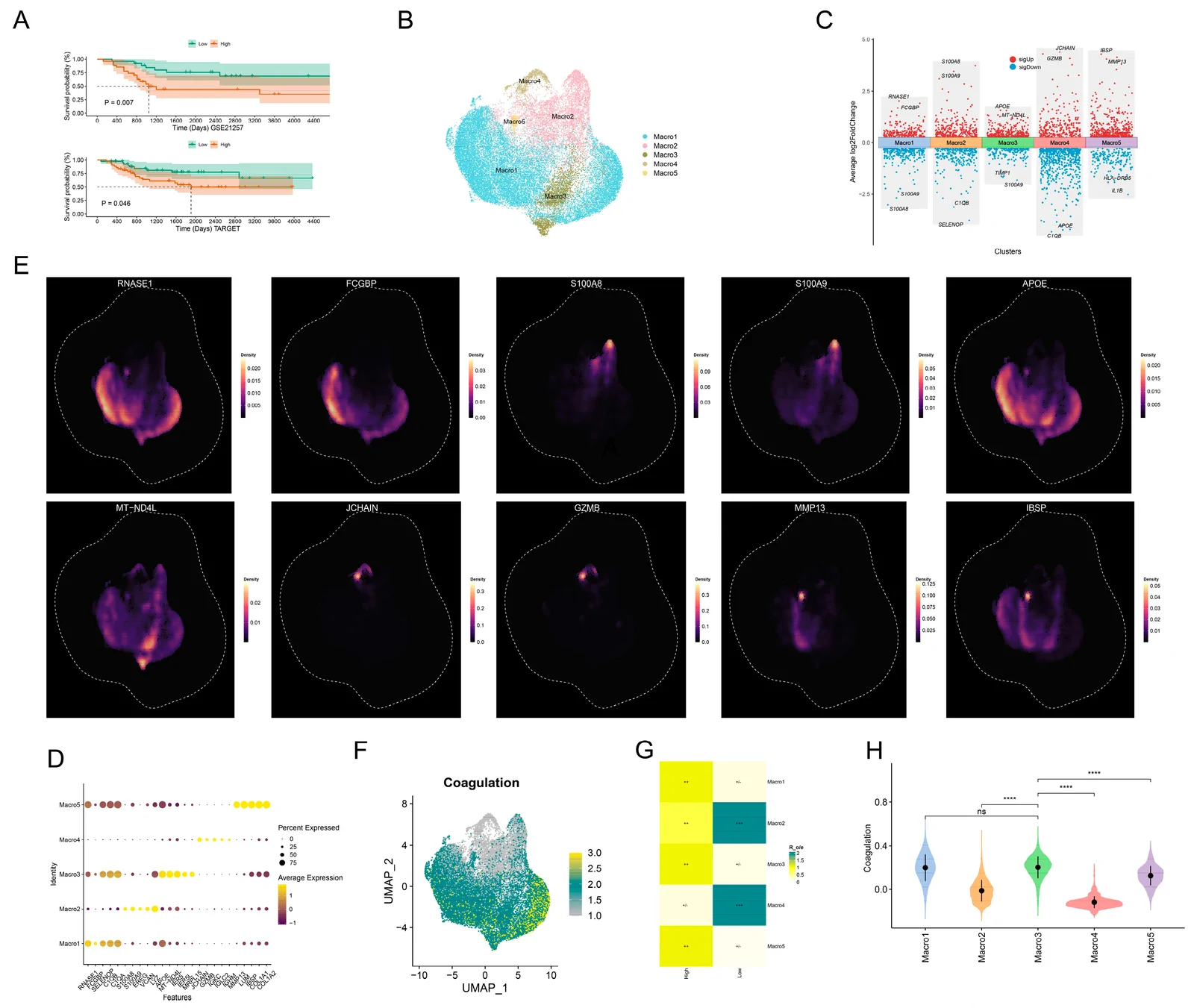
Macrophage heterogeneity and prognostic impact of coagulation-related marker genes in OS. (**A**) Prognostic Impact: The impact of the top 100 differentially expressed macrophage marker genes on the prognosis of OS patients. (**B**) Macrophages subgroups. (**C**,**D**) Marker Genes subgroups. (**E**,**F**) UMAP shows the distribution of macrophage coagulation scores. (**G**) Analysis of coagulation score preferences specific to each type of macrophage. Symbols denote relative cell distribution preference intensity: ‘+/−’ indicates weak/equivocal, ‘++’ moderate, and ‘+++’ strong preference. (**H**) Coagulation scores for each type of macrophage. ns, not significant; **** *p* < 0.0001.

Next, the characteristics of macrophages in OS were studied at single-cell resolution. Firstly, two single-cell data sets, GSE152048 and GSE162454, were preprocessed (Supplementary Figure S3A,B), and the number of gene expressions was 200 to 7500, and the proportion of mitochondrial genes was less than 20%, because if the single-cell gene expression is too low, they may be empty droplets; if the number of expressed genes is too high, they may be bicellular or multicellular; and if the mitochondrial genes are too high, this indicates that cells may be necrotic. The Harmony package was used to integrate GSE152048 and GSE162454 single-cell data sets and remove the batch effect. The results showed that the Harmony dimensionality reduction method is obviously better than the PCA method. According to the Cellmarker database and the related single-cell literature, cells were clustered into seven cell types, namely macrophages, osteoclasts, fibroblasts, NK/T cells, endothelial cells, B cells, and mast cells (Supplementary Figures S3C and S4A–C). The differentially up-regulated genes of the seven cell types were enriched in different pathways. There was a slight difference in the distribution of the two single-cell data sets on the UMAP map, which shows the intra-tumor heterogeneity in OS (Supplementary Figure S4D,E). In order to understand the transcription characteristics of coagulation-related genes at single-cell resolution, we used the 35 genes extracted by bulk RNA-seq to score and divided them into high and low groups according to the median coagulation score of all cells. There are obvious differences in the distribution of various types of cells in different coagulation score categories (Supplementary Figure S4F,G). Macrophages account for the largest proportion of immune cells in OS, indicating that they may play an important role in immune cell crosstalk in OS. In addition, the cell distribution preference analysis showed that macrophages were mainly in half of the cells with a high coagulation score (Supplementary Figure S4H). Interestingly, the distribution of the coagulation score and macrophages had more duplication, and macrophages had the highest coagulation score (Supplementary Figure S4I,J), and the top 100 differentially expressed genes of macrophages can significantly distinguish the prognosis of patients (Figure 3A). Consistently, clinical multivariable Cox regression analysis further validated the phenotypic impact of this hypercoagulable profile, demonstrating that pre-operative fibrinogen levels serve as a robust, independent risk factor for poor overall survival (HR = 1.894, 95% CI: 1.117–3.211, *p* = 0.0177) in patients with osteosarcoma (Supplementary Figure S5).

**Figure 4:**
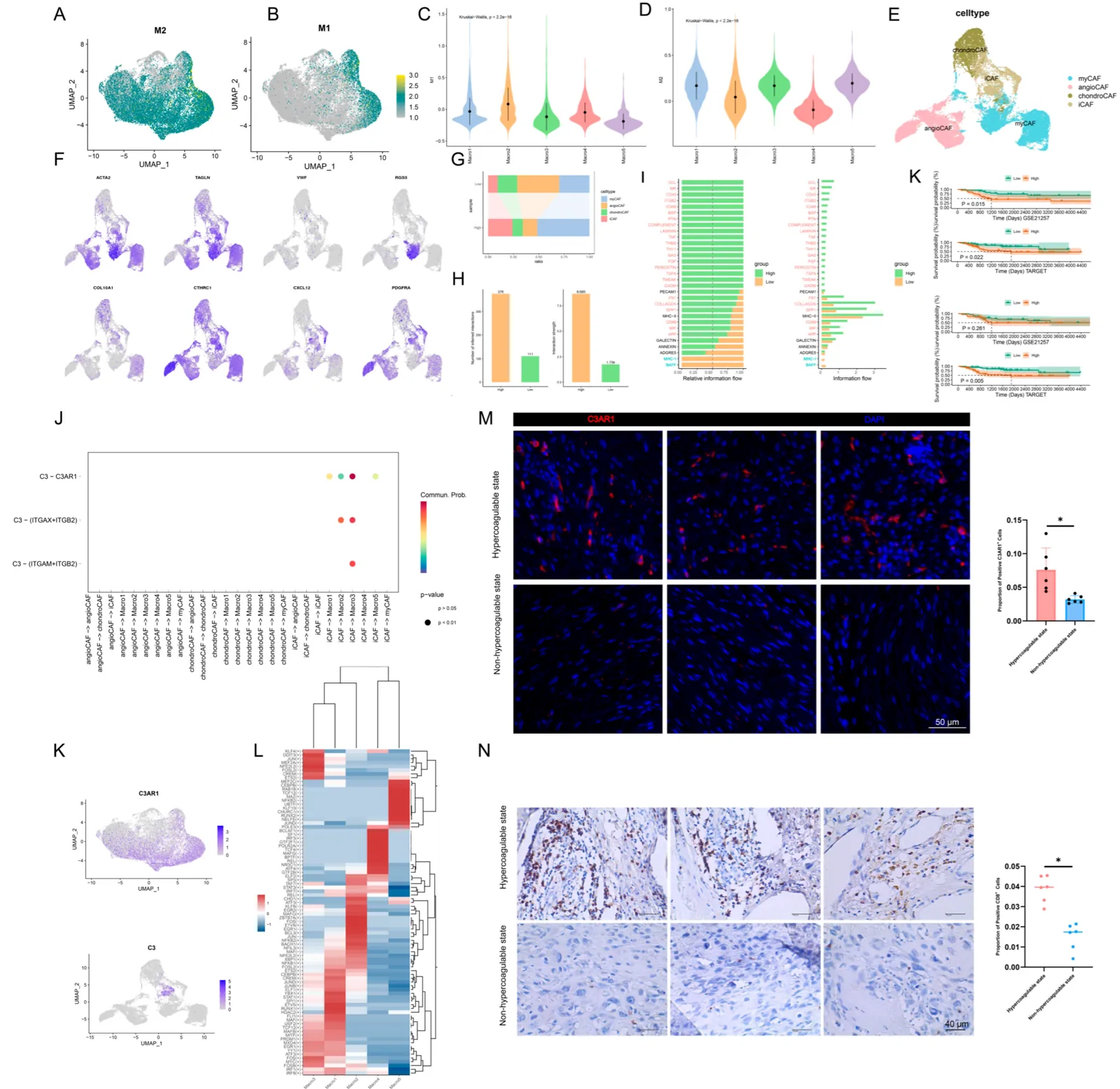
M1/M2 macrophage polarization dynamics and intercellular communication in coagulation-altered OS. (**A**–**D**) Distribution of M1 and M2 macrophage scores. (**E**,**F**) Subpopulation distribution proportions of fibroblasts and marker genes for each respective subpopulation. (**G**) Proportions of CAF subtypes (myCAF, angioCAF, chondroCAF, iCAF). (**H**,**I**) Pathways and quantity of intercellular communications in groups with different coagulation scores. (**J**) Cellular communication between macrophages and fibroblasts. (**K**) Relationship between the expression levels of *C3AR1* (**top**) and *CXCL12* (**bottom**) and prognosis in OS. (**L**) Transcriptional regulators specific to APOE^+^ macrophages. (**M**) IF images of OS tissue sections comparing C3AR1 expression in a hypercoagulable versus a non-hypercoagulable state. Results show markedly enhanced C3AR1 expression (red) in the high coagulation-related transcriptional score group. Nuclei were counterstained with DAPI (blue). Scale bars: 50 μm. (**N**) IH staining of C3AR1, in the hypercoagulable (**left**) and non-hypercoagulable (**right**) of patients with OS. Scale bars: 40 μm. Significant differences are indicated by * *p* < 0.05.

To elucidate the roles of coagulation-related genes and their transcriptional regulatory mechanisms within various macrophage subpopulations, we performed a subset analysis of macrophages. Among the five identified macrophage subpopulations (Figure 3B–E), three—Macro1, Macro3, and Macro5—were predominantly distributed in cells from the high coagulation score group (Figure 3F–H). Interestingly, these same subpopulations (Macro1, Macro3, and Macro5) exhibited higher levels of M2 macrophage activity (Figure 4A–D). M2-polarized TAMs suppress anti-tumor immune responses by secreting anti-inflammatory cytokines, such as IL-10 and TGF-β, thereby creating an immunosuppressive microenvironment that is conducive to tumor growth. Furthermore, M2 macrophages promote angiogenesis, as well as tumor cell migration and metastasis. The Macro3 subpopulation was characterized by high expression of *APOE*. Notably, a recent single-cell multi-omics study revealed that the proportion of APOE^+^ macrophages is higher in patients with triple-negative breast cancer who are non-responsive to immune checkpoint inhibitor (ICI) therapy. In murine models, the combination of an APOE inhibitor with ICI treatment demonstrated the most significant therapeutic efficacy. This suggests the potential therapeutic relevance of the Macro3 (APOE^+^ macrophage) subpopulation in cancer immunotherapy. To further characterize the functional heterogeneity of macrophage subpopulations, we observed that APOE^+^ macrophages resided within a hypoxic stromal niche. Notably, these cells concurrently upregulated immunosuppressive checkpoints and hypoxia-inducible pro-coagulant factors. Accumulating evidence suggests a tightly interconnected network between hypoxia signaling, coagulation activation, and immune modulation in the tumor microenvironment, where HIF-1–mediated hypoxic adaptation promotes both pro-coagulant states and immunosuppressive macrophage polarization [40] (Supplementary Figure S6A).

Given that numerous studies have reported that interactions between fibroblasts and macrophages can promote tumor progression, we conducted a subpopulation analysis of fibroblasts from the single-cell data. We annotated four fibroblast subpopulations: myCAFs, angioCAFs, chondroCAFs, and iCAFs (Figure 4E–G). Intriguingly, iCAFs were predominantly found in the high coagulation score subgroup, a distribution preference consistent with that of APOE^+^ macrophages. To investigate the interactions between fibroblasts and macrophages, we performed a cell–cell communication analysis, which revealed that intercellular communication was more active in the high coagulation score group (Figure 4H,I). Moreover, certain pathways, such as the COMPLEMENT pathway, were unique to the high coagulation-related transcriptional score group. We observed a interaction between iCAFs and APOE^+^ macrophages, primarily mediated by the C3–C3AR1 receptor-ligand pair (Figure 4J, Supplementary Figure S6B). Additionally, both *C3AR1* and the iCAF marker gene *CXCL12* were associated with a poorer prognosis in OS (Figure 4K). It has been reported in the literature that the C3–C3AR1 axis can promote M2 polarization of macrophages, which may explain the aforementioned higher levels of M2 polarization-related transcripts in APOE^+^ macrophages. To validate these findings, we used bulk RNA-seq data to explore the expression correlation between *C3*, *C3AR1*, and marker genes for iCAFs and APOE^+^ macrophages, all of which showed significant positive correlations.

Furthermore, the MAPK pathway was significantly enriched among the differentially upregulated genes in APOE^+^ macrophages. The RAF-MEK-ERK signaling cascade is a well-characterized MAPK pathway involved in cell proliferation and survival, suggesting that APOE^+^ macrophages may promote tumor progression via the MAPK pathway (Supplementary Figure S6C). A pySCENIC analysis identified the transcriptional regulons for each macrophage subpopulation, revealing that APOE^+^ macrophages have multiple significantly upregulated transcriptional regulators (Figure 4L, Supplementary Figure S6D). A growing body of literature has reported that c-Jun can promote tumor progression [41,42,43]. We validated the correlation between the C3–C3AR1 axis and APOE^+^ macrophage as well as M2 macrophage markers in the GSE21257 and TARGET-OS datasets. The results were consistent with our expectations, showing a significant positive correlation between the C3–C3AR1 axis and both APOE^+^ macrophages and M2 macrophage markers (Supplementary Figure S6E). Subsequently, we validated the expression of *C3AR1* in hypercoagulable and non-hypercoagulable states using immunohistochemistry and immunofluorescence. The results showed that *C3AR1* was highly expressed in the OS under the hypercoagulable state (Figure 4M,N). We quantified APOE^+^ macrophage abundance in multiple cohorts to validate the robustness of this macrophage state (Supplementary Tables). APOE^+^ macrophages appeared in all samples with variable but persistent abundance. Validation using the GSE299025 spatial transcriptomic dataset confirmed the presence of APOE^+^ macrophage-like signals within osteosarcoma tissue sections (Supplementary Figure S6F,G). A Venn diagram displays signal intersections among C3AR1, CD68, and APOE spots (Supplementary Figure S6H). The exclusive intersection of CD68 and APOE spots contains 3789 spots. We evaluated the composition of C3AR1-positive spots. CD68+ APOE+ macrophages represent 9.6 percent of these spots. Other microenvironments comprise 90.4% (Supplementary Figure S6I).

### 3.3. C3AR1 Is a Key Coagulation-Related Gene That Affects the Tumor Niche of OS

**Figure 5:**
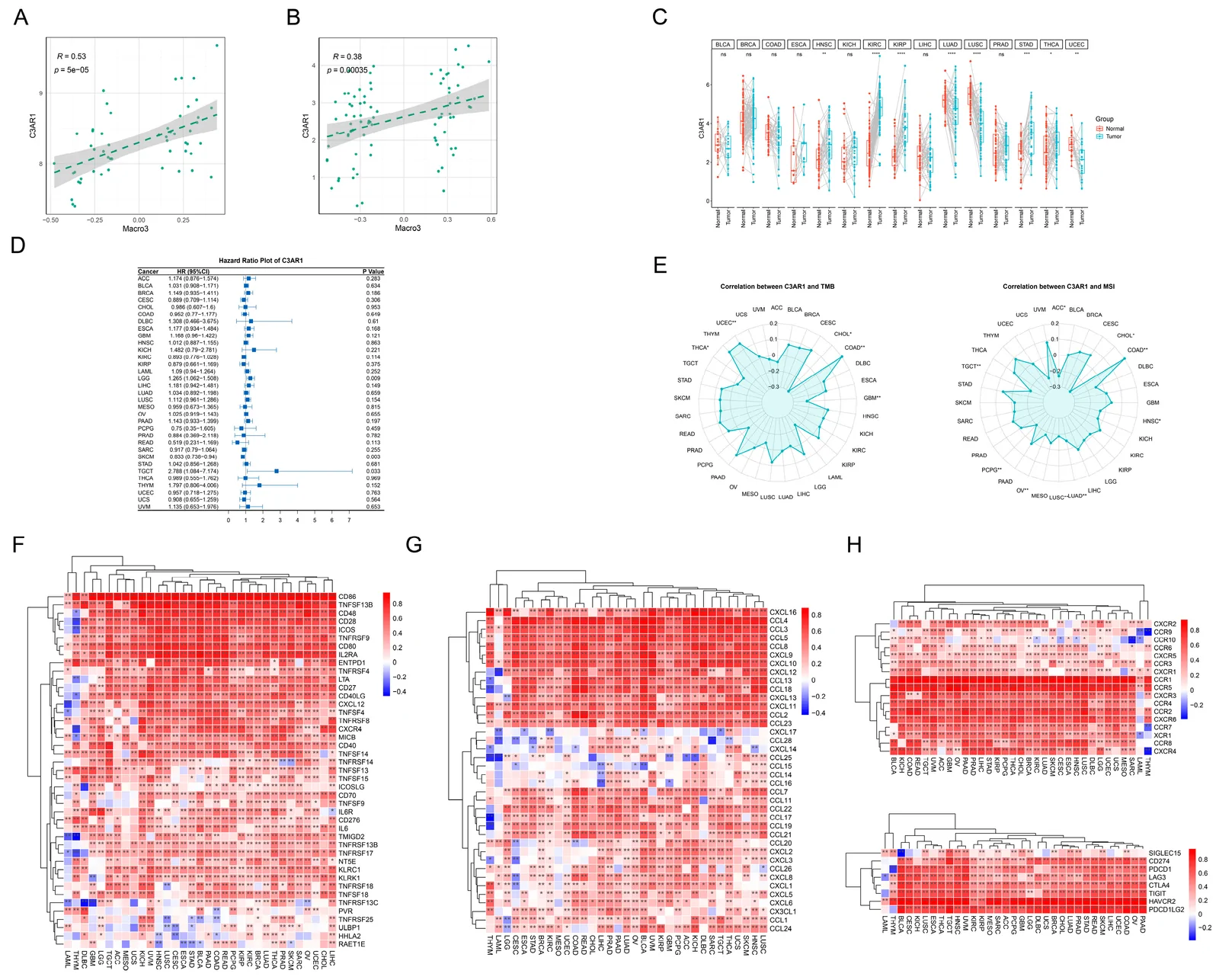
Pan-cancer analysis identifies *C3AR1* as a key coagulation-related oncogenic driver. (**A**) The scatter plot shows the correlation between *C3AR1* and differentially upregulated genes in Macro3 from the GSE21257 dataset. (**B**) The scatter plot shows the correlation between *C3AR1* and differentially upregulated genes in Macro3 from the target-OS dataset. (**C**) The TCGAPlot package is used to display the expression differences of *C3AR1* across various tumors and their corresponding normal tissues. (**D**) The forest plot of COX regression analysis shows the expression status of *C3AR1* in patients with different tumors. (**E**) The radar chart shows the correlation between *C3AR1* expression and TMB and MSI in different tumors. (**F**–**H**) The heatmap illustrates the correlation between *C3AR1* expression and cytokine receptors and ligands in different tumors. ns, not significant; * *p* < 0.05; ** *p* < 0.01; *** *p* < 0.001; **** *p* < 0.0001.

It was discussed that most coagulation-related genes were differentially expressed in OS, which affects and predicts the prognosis. In addition, coagulation-related genes are closely related to macrophages. Next, we focused on finding the coagulation-related genes that are most associated with APOE^+^ macrophages. Using the marker gene of APOE^+^ macrophage (the top 100 differentially up-regulated genes of APOE^+^ macrophage) and the expression of *C3AR1* to make correlation analysis, in the two datasets of GSE21257 and TARGET-OS, there was a significant positive correlation between the marker gene of APOE^+^ macrophage and the expression of *C3AR1* (*r* = 0.53, *p* = 5 × 10^−5^, Spearman; *r* = 0.38, *p* = 0.00035, Spearman, Figure 5A,B). To further support the transcriptomic inference from bulk and single-cell analyses, spatial transcriptomic data were leveraged to examine the in situ distribution pattern of C3AR1 and its cellular context. We observed that C3AR1 expression signals were predominantly localized within CD68^+^ macrophage-enriched regions, with substantial overlap with APOE^+^ macrophage niches. This spatial colocalization provides additional supportive evidence for a putative association between C3AR1 signaling and APOE^+^ macrophage populations in the osteosarcoma microenvironment.

### 3.4. Pan-Cancer Characterization of C3AR1

The TCGAPlot package is an R package for pan-cancer analysis and visualization of TCGA data, which can be used for pan-cancer expression and correlation analysis between gene expression and tumor mutational burden (TMB), Microsatellite instability (MSI), and promoter methylation. The results showed that *C3AR1* was significantly overexpressed in head and neck squamous cell carcinoma (HNSC), kidney renal clear cell carcinoma (KIRC), kidney renal papillary cell carcinoma (KIRP), lung adenocarcinoma (LUAD), lung squamous cell carcinoma (LUSC), stomach adenocarcinoma (STAD) and thyroid carcinoma (THCA) but significantly upregulated in uterine corpus endometrial carcinoma (UCEC) (Figure 5C). It showed that in most tumors, the related pathways involved in *C3AR1* are abnormally activated. COX regression analysis showed that *C3AR1* was significantly correlated with poor prognosis in testicular germ cell tumors (TGCT) and lower grade glioma (LGG), while it was significantly correlated with good prognosis in skin cutaneous melanoma (SKCM), indicating that *C3AR1* had obvious heterogeneity in different tumors (Figure 5D). Tumors with high TMB will produce more new antigens, which may effectively activate the immune system against tumors, such as inducing T lymphocyte infiltration by increasing the production of tumor new antigens. *C3AR1* was negatively correlated with TMB in glioblastoma multiforme (GBM), THCA, and cholangiocarcinoma (CHOL), suggesting that *C3AR1* may play a role in promoting tumor progression. Tumors with MSI-high usually respond well to immunotherapy. We found that, in ovarian serous cystadenocarcinoma (OV), USC, LUAD, HNSC, CHOL, adrenocoritical carcinoma (ACC), TGCT, and pheochromocytoma and paraganglioma (PCPG), *C3AR1* was significantly negatively correlated with MSI, which indicated that the results significantly negatively correlated with TMB led to similar results (Figure 5E). In addition, most of the expressions of *C3AR1* ligand with common immune checkpoints and chemokine receptors are significantly positively correlated (Figure 5F–H). For example, the interaction between programmed death-1 (PD1) and its ligand PD-L1 (programmed death ligand-1) leads to the functional exhaustion of T cells, thus helping tumor cells escape the attack of the immune system. This immune escape mechanism enables tumors to grow and spread. The types of chemokines and chemokine receptors are related to metastasis. Various chemokines and chemokine receptors are associated with metastasis, with the CXCL12/CXCR4 axis representing a key factor in this phenomenon, confirmed in multiple cancers.

## 4. Discussion

**Figure 6:**
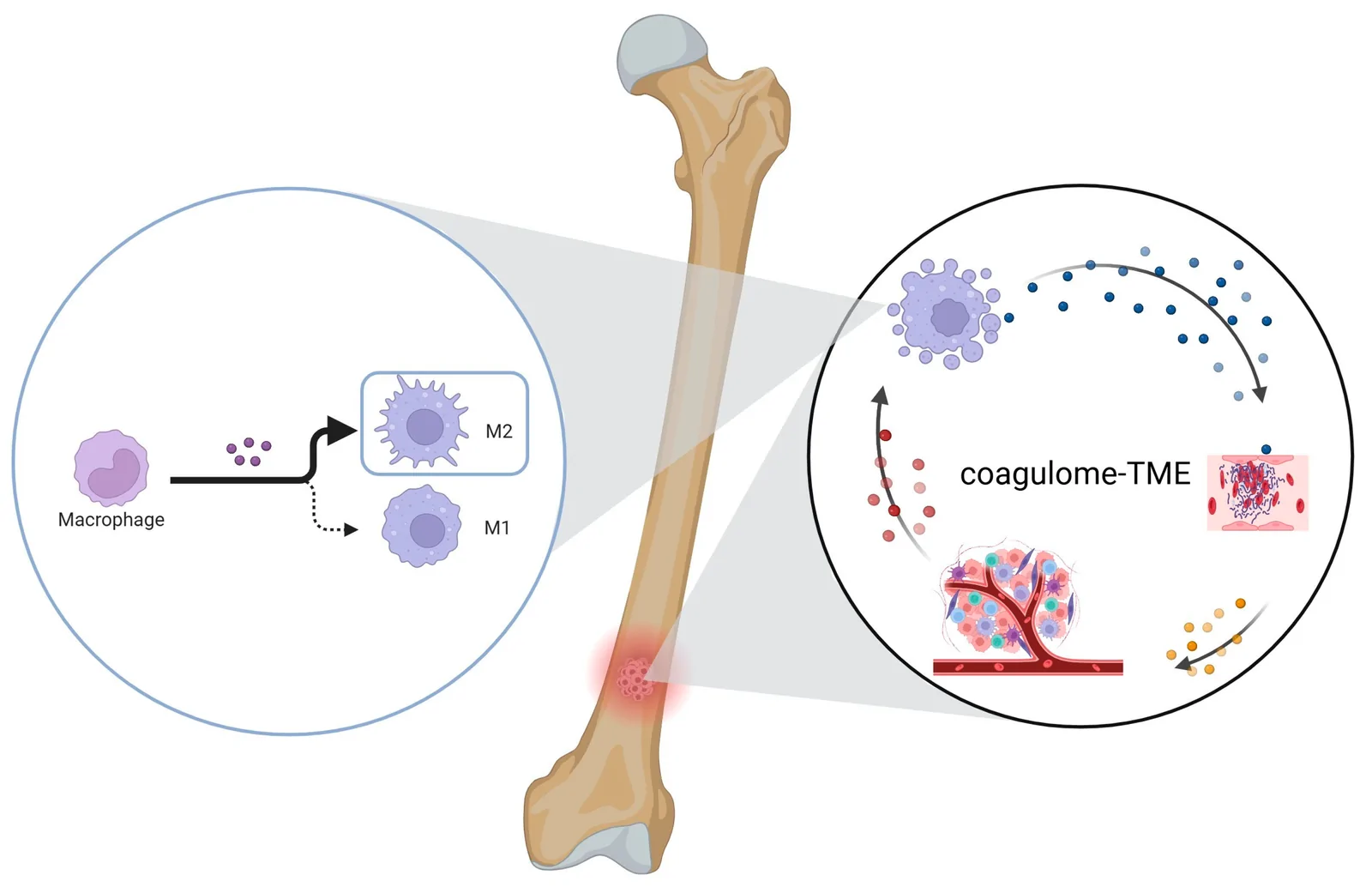
Interaction of coagulome-TME. Under a high coagulation-related transcriptional score group, iCAFs establish paracrine communication with APOE^+^ macrophages via the complement C3–C3AR1 signaling axis. This stromal–myeloid interplay critically drives the polarization of macrophages toward an immunosuppressive M2 phenotype. Ultimately, this coagulome-mediated network fosters a pro-tumorigenic niche, accelerating malignant progression and exacerbating adverse clinical outcomes.

Utilizing scRNA-seq and RNA-seq data, this study investigated the prognostic value of the tumor coagulome in OS and proposed a potential mechanism underlying tumor progression (Figure 6). Coagulation-related genes displayed expression heterogeneity that appeared correlated with patient survival. Single-cell analysis suggested that iCAFs and APOE^+^ macrophages were enriched in high coagulation score subgroups. Furthermore, cell–cell communication analysis indicated a potential interaction via the C3–C3AR1 axis. These findings imply that C3–C3AR1 signaling may drive M2 macrophage polarization, potentially facilitating OS progression.

Our results suggest that the activity level of coagulation-related transcriptional signatures in OS positively correlates with tumor malignancy, and the coagulation score holds independent predictive value for patient prognosis. The adverse prognostic impact of an active tumor coagulome in cancer patients has been widely reported across various malignancies, including lung cancer [44], breast cancer [45], prostate cancer [46], and pancreatic cancer [47]. For instance, elevated levels of fibrinogen (FIB) and D-dimer (DD) in prostate cancer have been confirmed to be associated with poor prognosis [46]. Currently, two main perspectives exist regarding the mechanism of action of the tumor coagulome. The first pertains to the direct pathological effects of cancer-associated thrombosis (CAT). Numerous studies have confirmed that malignant tumors activate the coagulation system, leading to venous/arterial thrombotic events such as deep vein thrombosis (DVT), pulmonary embolism (PE), or arterial thromboembolism (ATE). Such complications are not only life-threatening [48,49,50] but may also accelerate tumor progression through pro-inflammatory responses [51,52,53]. The second perspective focuses on the interaction between the tumor coagulome and the TME. A theoretical basis for this has been provided by the evolutionary link between the coagulation system and the complement cascade [54], as well as immune-coagulation crosstalk [6]. For example, the coagulation initiation complex (TF-FVIIa-FXa) can promote tumor immune evasion by inducing the chemokine CCL22 [55]. Our results show that OS samples with high coagulation scores exhibit significantly increased macrophage infiltration, and that APOE^+^ macrophages show actively upregulated coagulation-related transcriptional features, which extend and complement the second viewpoint. This study provides insights into the potential the molecular basis of coagulome-TME interaction, further supporting the notion that the tumor coagulome may be a major mechanism in OS pathogenesis and a potential therapeutic direction.

In the OS microenvironment, fibroblasts and macrophages form an intricate interaction network through various cytokines. They collectively contribute to an immunosuppressive microenvironment. This niche promotes tumor progression and therapeutic resistance. This interaction is particularly prominent in the high coagulation score subgroup of OS. Within this subgroup, iCAFs may facilitate the M2 polarization program of APOE^+^ macrophages via a candidate C3–C3AR1-associated interaction. This crosstalk potentially accelerates the malignant progression of the tumor. This mechanism suggests potential pan-cancer applicability. Complement C3 is aberrantly overexpressed in multiple tumor types. It is associated with immune evasion and therapeutic resistance [56]. For instance, fibroblasts and macrophages establish a stromal-myeloid niche via C3–C3AR1 signaling in a model of gastric cancer peritoneal metastasis [57]. This specific niche leads to resistance to immune checkpoint blockades. In glioblastoma, C3a directly induces the transformation of microglia and macrophages toward an M2 phenotype [58]. This process occurs in a C3aR-dependent manner. Furthermore, research has identified C3a/C3aR signaling as a central hub for crosstalk between the complement system and macrophages [59]. This pathway acts as a key driver of immunosuppression in sarcoma. These published findings are highly consistent with our transcriptomic results. They collectively identify this candidate interface as a potential therapeutic target [60]. Moreover, our pan-cancer analysis supports the pro-tumorigenic role of C3AR1. This finding further validates its broad applicability across multiple cancer types. To our knowledge, this study is the first to propose a model of microenvironmental remodeling in OS. This remodeling involves a candidate C3–C3AR1–APOE^+^ macrophage interaction framework. This model provides a novel target for combination therapies against this specific stromal–immune crosstalk. However, gene-level transcription does not equal functional protein activation [61]. Increased C3 expression does not guarantee proteolytic cleavage of the C3 precursor protein [62]. Similarly, C3AR1 up-regulation does not confirm downstream receptor signaling [63]. Our study did not directly measure local fluid concentrations of C3a. We also did not evaluate the phosphorylation status of the C3a receptor. Therefore, the actual generation of C3a, and its receptor activation cannot be verified at this stage.

In the OS microenvironment, fibroblasts and macrophages form an intricate interaction network through various cytokines, collectively contributing to an immunosuppressive microenvironment that promotes tumor progression and therapeutic resistance. This study finds that this interaction is particularly prominent in the high-coagulation score subgroup of OS. Within this subgroup, iCAFs may facilitate the M2 polarization program of APOE^+^ macrophages via the complement C3–C3AR1 axis, thereby accelerating the malignant progression of the tumor. This mechanism suggests potential pan-cancer applicability. Complement C3 is aberrantly overexpressed in multiple tumor types and is associated with immune evasion and therapeutic resistance [56]. For instance, in a model of gastric cancer peritoneal metastasis, fibroblasts and macrophages establish a stromal-myeloid niche via the C3–C3AR1 axis, leading to resistance to ICBs [57]. In glioblastoma, C3a directly induces the transformation of microglia and macrophages toward an M2 phenotype in a C3aR-dependent manner [58]. Furthermore, research has identified C3a/C3aR signaling as a central hub for crosstalk between the complement system and macrophages, confirming it as a key driver of immunosuppression in sarcoma [59]. These published findings are consistent with our results, collectively suggesting that the C3–C3AR1 axis is a potential therapeutic target. Moreover, our pan-cancer analysis supports the pro-tumorigenic role of *C3AR1*, further validating its broad applicability across multiple cancer types. To our knowledge, this study is the first to propose a model in OS where iCAFs may remodel the immune microenvironment via a C3–C3AR1–APOE^+^ macrophage axis, thereby providing a novel target for combination therapies aimed at disrupting this stromal–immune crosstalk.

This study has several limitations that should be acknowledged. First, it is primarily an in silico investigation integrating bulk and single-cell transcriptomic datasets with retrospective clinical data, and although supported by preliminary histological validation, it lacks comprehensive functional experiments to establish causality. Second, the coagulation-related transcriptional signature reflects pathway activity derived from complement and coagulation gene sets at the transcriptomic level and does not directly represent clinical hypercoagulability; therefore, its interpretation may be confounded by tumor cellular composition, particularly macrophage enrichment. Third, despite multi-cohort integration, differences in sequencing platforms, data processing pipelines, and tissue sources may introduce residual batch effects that cannot be fully eliminated by statistical correction. Fourth, single-cell analyses may be influenced by inter-sample heterogeneity, and certain macrophage or stromal subpopulations may be disproportionately driven by individual samples, limiting generalizability. Fifth, ligand–receptor communication inferred by CellChat represents putative interactions based on transcript abundance and curated databases, and does not confirm actual ligand secretion or receptor activation; thus, the proposed C3–C3AR1 axis should be interpreted as a candidate signaling pathway rather than a validated mechanistic link. Finally, the limited sample size and event number in clinical cohorts constrain the robustness of multivariate survival models and increase the risk of overfitting, while incomplete adjustment for potential confounders may introduce residual bias. Therefore, all findings should be considered hypothesis-generating and require further validation in larger, prospective cohorts and experimental systems.

## 5. Conclusions

In summary, this study investigated the prognostic implications of the tumor coagulome in OS and characterized putative interactions with APOE^+^ macrophages. Our analysis highlights C3AR1 as a candidate coagulation-related gene that may contribute to OS pathogenesis. Collectively, these observations offer fresh perspectives that could potentially inform future therapeutic strategies.

## Figures and Tables

**Table 1:** Patient baseline demographic and clinical characteristics.

Characteristic	Overall N = 59	Survival N = 41	Death N = 18	*p*-Value
White Blood Cell Count, (×10^9^/L)	9.616 ± 9.037	9.408 ± 8.532	10.088 ± 10.345	0.576
Absolute Neutrophil Count, (×10^9^/L)	6.817 ± 7.036	6.999 ± 7.248	6.424 ± 6.740	0.817
Absolute Lymphocyte, (×10^9^/L)	1.548 ± 0.689	1.496 ± 0.650	1.669 ± 0.778	0.556
Absolute Monocyte Count, (×10^9^/L)	0.603 ± 0.557	0.626 ± 0.572	0.545 ± 0.529	0.636
Absolute Eosinophil Count, (×10^9^/L)	0.087 ± 0.107	0.098 ± 0.120	0.060 ± 0.059	0.290
Absolute Basophil Count, (×10^9^/L)	0.057 ± 0.151	0.034 ± 0.027	0.107 ± 0.270	0.760
Triglycerides, (mmol/L)	1.334 ± 0.895	1.295 ± 0.695	1.428 ± 1.277	0.370
Cholesterol, (mmol/L)	3.898 ± 1.074	3.986 ± 1.179	3.697 ± 0.775	0.639
HDL-C, (mmol/L)	1.171 ± 0.374	1.226 ± 0.391	1.046 ± 0.307	0.152
LDL-C, (mmol/L)	2.204 ± 0.833	2.272 ± 0.916	2.049 ± 0.594	0.687
PT, (s)	12.076 ± 3.167	11.744 ± 0.951	12.833 ± 5.587	0.941
INR, (ratio)	1.017 ± 0.075	1.017 ± 0.080	1.016 ± 0.066	0.908
aPTT, (s)	29.125 ± 3.834	28.920 ± 3.607	29.594 ± 4.382	0.711
TT, (s)	18.566 ± 2.838	18.327 ± 3.225	19.111 ± 1.599	0.173
Fibrinogen, (g/L)	3.215 ± 0.817	3.085 ± 0.800	3.511 ± 0.800	0.045
Gender, n (%)				0.133
Female	25 (42%)	20 (49%)	5 (28%)	
Male	34 (58%)	21 (51%)	13 (72%)	
Age, (years)	21.305 ± 13.316	20.707 ± 12.528	22.667 ± 15.259	0.921
Enneking stage, n (%)				0.813
IIA	1 (2%)	1 (3%)	0 (0%)	
IIB	55 (93%)	39 (93%)	11 (91%)	
III	3 (5%)	2 (5%)	1 (9%)	
Pathological Fracture, n (%)	6 (10%)	3 (7.3%)	3 (17%)	0.415
Recurrence, n (%)	28 (47%)	10 (24%)	18 (100%)	<0.001
Metastasis, n (%)	6 (10%)	1 (2.5%)	5 (28%)	0.044
OS, (days)	1232.712 ± 497.661	1414.049 ± 348.596	819.667 ± 546.905	<0.001

## Data Availability

The original contributions presented in this study are included in the article/Supplementary Materials. Further inquiries can be directed to the corresponding authors.

## Supplementary Materials

The following supporting information can be downloaded at: https://www.mdpi.com/article/10.3390/biomedicines14081686/s1, Supplementary Figure S1. Characterization of coagulation genes and quality control of single-cell datasets. A–B. GSE21257 and TARGET-OS datasets feature 35 key coagulation genes. On the left are various metrics of each gene in random forest analysis; on the right is the correlation analysis between coagulation scores and different tumor characteristics. Supplementary Figure S2. Association between coagulation scores and immune microenvironment landscapes. A. The heatmap shows the correlation between coagulation scores and HALLMARKER50 in the GSE21257 and target-OS datasets. B. The heatmap shows the correlation between coagulation scores and tumor-associated functional gene sets in the GSE21257 and target-OS datasets. datasets. C–D. Immune cell abundance in GSE21257 and target-OS using the xCELL and EPIC methods. datasets. E–F. Differences in macrophage, M1 macrophage, and M2 macrophage abundance among different coagulation score groups. Supplementary Figure S3. A. Quality control results of single-cell datasets from GSE152048 and GSE162454. B. Harmonize and integrate single-cell datasets from GSE152048 and GSE162454 to remove batch effects. C. Density maps of different cell types in the single-cell dataset. Supplementary Figure S4. Single-cell atlas of OS reveals cell-type-specific coagulation score distribution. A. UMAP of the combined GSE152048 and GSE162454. B. single-cell datasets. C–D. Marker genes for seven cell types. D. Summary of the proportion of each cell type in the single-cell dataset. E. Distribution of the GSE152048 and GSE162454 datasets in the UMAP. F. Preference analysis of clotting scores for various cell types. G. Distribution of clotting scores. H. Representative proportions of each cell type in different clotting score groups. I. Clotting scores for various cell types. J. Distribution of macrophages. Supplementary Figure S5. Dual-panel forest plot representing univariate screening and multivariate-adjusted Cox regression analyses of clinical and hematological variables for overall survival in the osteosarcoma cohort (N = 59). Supplementary Figure S6. A. Heatmap displaying the relative activation scores of fifteen myeloid-specific biological pathways across five distinct macrophage subpopulations. B. Expression correlation of key genes within the C3-C3AR1 axis. C. Significant upregulation of the MAPK pathway among the differentially upregulated genes in APOE+ macrophages. D. Transcription factors of macrophage subtypes in the high coagulation-related transcriptional score group. E. The correlation between the C3-C3AR1 axis and APOE+ macrophages and M2 macrophage markers in the GSE21257 and TARGET-OS datasets. F. In situ tissue distribution and transcript density mapping of C3AR1, CD68, and APOE across the human osteosarcoma sections. G. UMAP visualization: Spot-level unsupervised clustering and deconvolution identifying major microenvironmental lineages, including macrophages and fibroblast subpopulations (iCAFs/myCAFs). H. Venn diagram intersection: Quantitative overlap analysis showing the absolute transcriptomic spot intersection counts among C3AR1_Spots, CD68_Spots, and APOE_Spots. I. Conditional localization bar plot: Mathematical probability distribution demonstrating that 9.6% (n = 54) of all captured C3AR1+ signals are strictly confined within the CD68+APOE+ macrophage niche. Supplementary Table: Table S1. Details of the public data set used in this article. Table S2. Single-cell RNA-seq datasets and preprocessing information. Table S3. Key computational parameters for scRNA-seq integration and clustering. Table S4. Demographic characteristics of TARGET-OS. Table S5. Demographic characteristics of GSE21257. Table S6. Demographic characteristics of GSE152048. Table S7. Demographic characteristics of GSE299025. Table S8. Characteristics of the 6 patients with OS included in GSE162454. Table S9. Candidate genes selected by LASSO regression and their correlation coefficients. Table S10. Differential expression matrix of the 35 coagulation-related DEGs. Table S11. Cell number of APOE+ and iCAF Macrophage in each sample. Table S12. Multivariable Cox regression model: Coagulation score and Macrophage abundance. Table S13. Immunosuppression gene set, Inflammatory regulation gene set. Table S14. Inflammatory regulation gene set. Table S15. Tissue repair gene set. Table S16. Phagocytosis gene set. Table S17. Lipid metabolism gene set. Table S18. Macrophage subtype-specific marker genes. Table S19. Cell-type marker genes in GSE299025. Table S20. Cell-type marker genes in the osteosarcoma scRNA-seq atlas. Table S21. Functional gene set for scoring. Table S22. Proportion of Positive C3AR1+ Cells. Table S23. Proportion of Positive CD8+ Cells.
